# Long-Term Follow-Up into Adulthood of Pediatric-Onset Primary Sclerosing Cholangitis and Autoimmune Sclerosing Cholangitis

**DOI:** 10.1097/PG9.0000000000000220

**Published:** 2022-06-21

**Authors:** Julian Hercun, Philippe Willems, Marc Bilodeau, Catherine Vincent, Fernando Alvarez

**Affiliations:** From the * Liver Unit, Centre hospitalier de l’Université de Montréal, Montréal, Canada; † Department of Gastroenterology, Hepatology and Nutrition, Centre Hospitalier Universitaire Sainte-Justine, Montréal, Canada.

**Keywords:** autoimmune liver disease, pediatric liver disease, liver transplantation

## Abstract

Studies on pediatric patients with primary sclerosing cholangitis (PSC) have been limited by short follow-up and inconsistent classification of pediatric patients with autoimmune hepatitis-sclerosing cholangitis overlap (AIC). We conducted a retrospective study of patients diagnosed with AIC or PSC during childhood with extension of follow-up into adulthood.

**Methods:**

We reviewed records of patients followed for PSC or AIC between 1998 and 2019 at a pediatric referral center. Features at diagnosis, biochemical and liver-related outcomes (cholangitis, liver transplant, and cirrhosis) were compared.

**Results:**

Forty patients (27 PSC, 13 AIC) were followed for 92 months on average (standard deviation 79 months) with extension into adulthood in 52.5%; 70% had associated inflammatory bowel disease (IBD). The proportion of patients with significant fibrosis and abnormal baseline liver tests (serum bilirubin and transaminase levels) were similar in both groups. One year postdiagnosis, 55% (15/27) of PSC patients had normal liver tests versus only 15% (2/13) in the AIC group (*P* = 0.02). During follow-up, more liver-related events occurred in the AIC group (69% versus 27%, hazard ratio [HR] = 3.7 [95% confidence interval (CI): 1.4–10] *P* = 0.01). Baseline elevated serum bilirubin levels (HR = 5.3 [95% CI: 1.7–16.9] *P* = 0.005) and elevated transaminase levels at 1 year (HR = 9.09 [95% CI: 1.18–66.7) *P* = 0.03) were predictive of liver-related events, while having IBD was not (HR = 0.48 (95% CI: 0.15–1.5) *P* = 0.22).

**Conclusions:**

Pediatric patients with AIC and PSC presented at a similar fibrosis stage, however, with a more severe hepatitis in AIC. In this cohort, AIC was associated with more liver-related events, primarily driven by a higher rate of cirrhosis compared with PSC; transplant rates were similar.

**What Is Known**

- Whether autoimmune hepatitis-sclerosing cholangitis overlap (AIC) represents a disease phenotype with a distinct natural history from primary sclerosing cholangitis (PSC) remains unclear.
- Long-term outcomes of pediatric patients with sclerosing cholangitis into adulthood have not been extensively studied.

**What Is New**

- In this single-center study, pediatric patients with PSC and AIC presented at a similar stage of fibrosis; however, synthetic liver dysfunction at presentation was more common in AIC.
- Diagnosis of AIC, elevated baseline bilirubin level, and persistently abnormal transaminase levels were associated with more liver-related adverse outcomes (primarily cirrhosis) on long-term follow-up.

## INTRODUCTION

Primary sclerosing cholangitis (PSC) is a cholangiopathy of unclear etiology characterized by progressive inflammation and fibrosis of the intra and/or extrahepatic bile ducts. PSC has an estimated prevalence of 1.5/100 000 children in North America (1). No effective treatment has been shown to alter its natural history. PSC is an important risk factor for recurrent bacterial cholangitis and can progressively lead to cirrhosis, portal hypertension, and end-stage liver disease requiring liver transplantation (LT) (2–5). While adult-onset PSC has been historically associated with a grim prognosis due to a high risk of developing liver failure or bile duct cancer, pediatric-onset autoimmune biliary disease potentially has a different, more favorable, prognosis (2).

PSC has been strongly associated with other autoimmune diseases, particularly inflammatory bowel disease (IBD), which has been reported in up to 80% of patients in some cohorts (2,6). The most common IBD phenotype in pediatric-onset PSC is a mild pancolitis and clinical symptoms do not correlate with endoscopic and histologic activity (7,8). PSC can also present with overlap features of autoimmune hepatitis such as elevated serum IgG and characteristic autoantibodies, and the presence of portal and lobular inflammation with piecemeal necrosis on histology. This phenotype is referred to as autoimmune cholangitis (AIC), autoimmune hepatitis overlap (PSC-AIH), or autoimmune sclerosing cholangitis (ASC) (9). few data are published on the influence of these phenotypes on the natural history of PSC. AIC has been associated with better prognosis, possibly because of the beneficial effect of administered immunosuppression, but larger cohorts have shown contradictory results (2,10,11). Concomitant IBD has also been shown to be associated with a better prognosis, perhaps for the same reason (2,6). However, most of the studies are limited by short follow-up period, inconsistent classification of AIC and incomplete information on immunosuppression. Whether AIC represents a disease phenotype with a distinct natural history from PSC remains unclear.

The aims of this study were to assess the long-term evolution of patients diagnosed with PSC and to test the hypothesis that phenotype, when rigorously defined, can help better determine the outcomes of autoimmune biliary disease. Our secondary aim was to see how response to immunosuppressive therapy influences the progression of PSC.

## METHODS

All patients followed for sclerosing cholangitis at the hepatology clinic at the Centre Hospitalier Universitaire (CHU) Sainte-Justine in Montreal, Canada, a pediatric referral center, between January 1998 and December 2018 were evaluated retrospectively. Patients were included if they were under 18 years of age at time of initial presentation and had a diagnosis of sclerosing cholangitis. Exclusion criteria were secondary sclerosing cholangitis (due to ischemia, trauma, infectious causes, post-LT, or an underlying condition associated with secondary sclerosing cholangitis) or a history of concomitant liver disease (with the exclusion of autoimmune hepatitis) or LT before diagnosis. Cholangiographic findings available for analysis were obtained from either magnetic resonance cholangiography, endoscopic retrograde cholangiography, or percutaneous cholangiography. Patients were classified by study investigators as either having AIC (evidence of large duct disease on cholangiography with histological features consistent with autoimmune hepatitis) or PSC (evidence of large duct disease on cholangiography or histological features consistent with small duct disease without evidence of autoimmune hepatitis). Findings of interface hepatitis, portal lymphoplasmacytic infiltrates, and hepatic rosette formation were considered consistent with autoimmune hepatitis, while neoductular proliferation and periductal concentric fibrosis were considered consistent with sclerosing cholangitis (12)

Baseline laboratory, histological, and imaging characteristics were recorded at the time of the initial evaluation or referral. The METAVIR system was used to stage fibrosis (13) and histology was reviewed with a pathologist if specific findings were not described. Upper limits of normal values in use at the CHU Sainte-Justine laboratory were used for analysis. Cirrhosis was determined by findings on imaging (nodular liver and splenomegaly) and/or compatible histological analysis. Treatment at baseline was defined as medications already in use at the time of diagnosis as well as medications initiated within 3 months after diagnosis. Early use of biologic agents (antitumor necrosis factor-alpha [anti-TNF] agents) was defined as introduction within 12 months following the diagnosis.

Laboratory values one year after the initial evaluation and clinically significant events (defined as a diagnosis of cirrhosis, neoplasia, LT, or any episode of cholangitis) in the longitudinal follow-up were collected. Upon reaching adulthood, patients were transitioned to the liver clinic of an adult hepatology referral center, the Centre hospitalier de l’Université de Montreal (CHUM). Clinical events occurring until May 2020 were included in the long-term follow-up.

Continuous variables were analyzed using t-tests or appropriate nonparametric tests and categorical variables using Chi square or Fisher’s exact test. Long-term outcomes and liver-related events, taking under account time to event, were evaluated through Cox proportional hazards regression analysis. Patients not meeting clinical endpoints were censored at time of last follow-up. *P* values <0.05 were considered significant. Statistical analysis was performed using R (version 3.6.1) and Graphpad Prism (version 8.4.1).

This project was approved by the Research Ethics Committees of the CHU Sainte-Justine (Review number 2018-1842) and the CHUM (Review number 20.062).

## RESULTS

### Baseline Characteristics

We identified 40 pediatric patients with sclerosing cholangitis, 27 classified as having PSC and 13 AIC. Overall, 62.5 % of patients were male, average age at initial presentation was 11.6 years (range 2–17 years of age). Of the PSC patients, 81.5% (22/27) had large duct disease on imaging with the residual 18.5% (5/27) presenting with small duct disease solely on histology. Baseline characteristics for the entire cohort are presented in Table 1.

**TABLE 1.** Baseline characteristics.

	Overall	PSC (N = 27)	AIC (N = 13)	*P*
Male sex, % (n)	62.5% (25)	59.3% (16)	69.2% (9)	0.73*
Age at diagnosis (SD)	11.6 (3.6)	10.85 (3.78)	13.15 (2.88)	0.06†
Follow-up (months) (SD)	92 (79)	107 (88)	60 (43)	0.08†
Associated IBD, % (n)	70% (28)	77.8% (21)	53.8% (7)	0.15‡
UC, % (n)	39.3% (11)	29.6% (8)	23.1% (3)	1.0‡
CD, % (n)	39.3% (11)	29.6% (8)	23.1% (3)	1.0‡
IC, % (n)	21.4% (6)	18.5% (5)	7.7% (1)	0.63‡
Presentation of liver disease in IBD (n = 28)				
At diagnosis of IBD, % (n)	30.4% (13)	47.6% (10)	42.8% (3)	0.99‡
In known IBD patient, % (n)	42.8% (12)	47.6% (10)	28.6% (2)	0.66‡
Before IBD diagnosis, % (n)	10.7% (3)	4.8% (1)	28.6% (2)	0.15‡
Significant fibrosis on biopsy (N = 32), % (n)	53.1% (17)	45.0%(9)	66.7%(8)	0.23*
Stigmata of cirrhosis on imagery (N = 31), % (n)	19.4% (6)	10.0% (2)	36.4% (4)	0.15‡
Total bilirubin (µmol/L)	18.8 (34.5)	16.3(38.0)	23.81(26.9)	**0.04** †
GGT (U/L)	236 (199)	236 (218)	237 (167)	0.59†
AST (U/L)	118 (120)	106(128)	145 (102)	0.09†
ALT (U/L)	170 (158)	147 (142)	216 (184)	0.15†
ALP (U/L)	390 (240)	374 (213)	421 (290)	0.71†
IgG (g/L)	18.9 (7.6)	15.3 (5.7)	24.7 (6.6)	**0.0005** †
Platelet counts (× 10^9^/L)	331 (136)	369 (109)	255 (154)	0.05†
INR	1.11 (0.18)	1.05 (0.07)	1.20 (0.26)	**0.008** †
Albumin (g/dL)	3.6 (0.6)	3.7 (0.6)	3.4 (0.7)	0.15†
ANA (≥1:40), % (n)	37.5% (15)	29.4% (5)	83.3% (10)	**0.004** ‡
Anti-SM (≥1:40), % (n)	7.5% (3)	0% (0)	33.3% (3)	**0.02** ‡
Anti-LKM (≥ 1:40)	0	0	0	-

All continuous values are expressed as means (standard deviation) unless specified otherwise. Categorical values expressed as percentages. Upper limit of normal: Total bilirubin: 17 µmol/L, ALT: 30 U/L, AST: 40 U/L, GGT: 25 U/L, Albumin: 3.5 g/L, Platelet counts: 150 × 10^9^/L, IgG: 15g/L. ALP normal levels varied by age. AIC = autoimmune cholangitis; ALP = alkaline phosphatase; ALT = alanine transaminase; ANA = antinuclear antibody; AST = aspartate transaminase; Anti-LKM = anti-liver kidney microsome antibody; Anti-SM = anti-smooth muscle antibody; CD = Crohn’s disease; IBD = inflammatory bowel disease; IC = indeterminate colitis; GGT = gamma-glutamyl transferase; PSC = primary sclerosing cholangitis; SD = standard deviation; UC = ulcerative colitis.

*Chi squared.

†Wilcoxon rank sum test.

‡Fisher’s exact test.

Bold indicates significant of *P* < 0.05.

**FIGURE 1.**
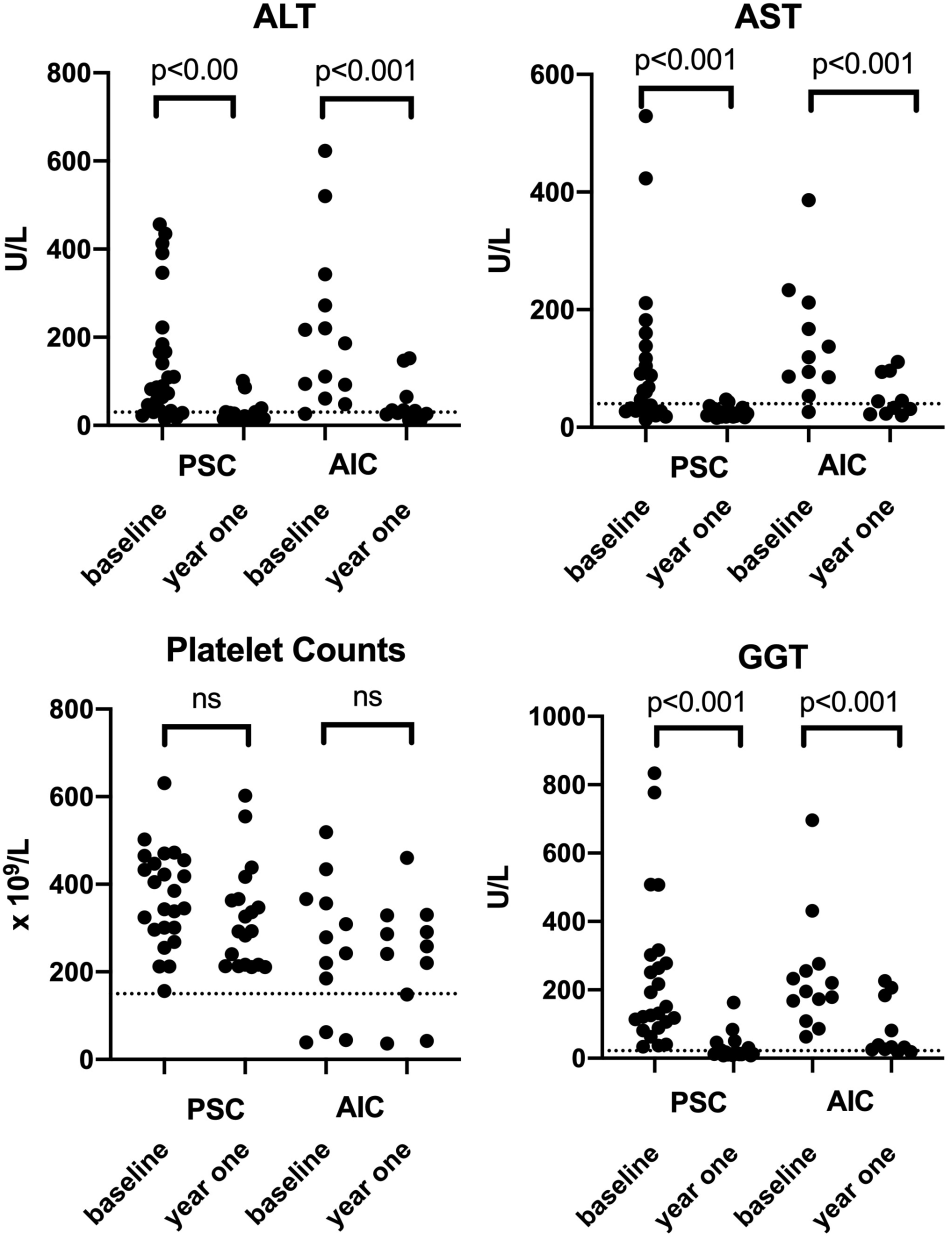
Comparison of laboratory values at baseline and at 1 year. All *P* values are Wilcoxon rank sum tests. AIC = autoimmune cholangitis; ALT = alanine transaminase; AST = aspartate transaminase; GGT = gamma-glutamyl transferase; PSC = primary sclerosing cholangitis.

Liver disease was diagnosed at the same time as diagnosis of IBD in 32.5% of patients (13/40), whereas in 30% (12/40) liver disease was diagnosed in patients with established IBD. In addition, 7.5% of patients (3/40) had liver disease diagnosed before appearance of IBD symptoms. While a majority of patients had no symptoms of liver disease at initial presentation (no pruritus or jaundice), 17.5% (7/40) presented with elevated transaminase levels (38.5% in AIC versus 7% in PSC, *P* = 0.03) and 5% (2/40) with symptomatic cholestasis (8% in AIC versus 4% in PSC, *P* = ns). While most laboratory test results were comparable between both groups, IgG, autoantibodies, INR, and bilirubin were significantly higher in the AIC group, while platelet counts were lower in the AIC group. Elevated IgG levels were associated with AIC; however, this did not remain significant in the presence of autoantibodies (see Supplemental Digital Figure 1, http://links.lww.com/PG9/A86, Receiver Operative Characteristic curves).

Liver biopsy was performed in 32 patients at diagnosis. In the 13 AIC patients, 92% had interface hepatitis, 62% neoductular proliferation and 38% periductal concentric fibrosis. In the PSC subgroup, 5% had interface hepatitis, 55% neoductular proliferation, and 55% periductal concentric fibrosis. The average fibrosis stage was 2, with 53% (17/32) of patients presenting with significant fibrosis at diagnosis. Presence of significant fibrosis (>F2) was similar between PSC (45%) and AIC (67%) (*P* = 0.23).

Overall, 70% (28/40) had associated IBD with an equal percentage of Crohn’s disease and ulcerative colitis (39.3%) and 21.4% presenting with indeterminate colitis. IBD was numerically but not statistically more common in patients with PSC (77.8% PSC and 53.8% AIC, *P* = 0.15). Overall, 5% of patients (2/40) had another concomitant nongastrointestinal autoimmune disease.

### Pharmacological Treatment

Eighty percent of patients (32/40) were treated from the onset with ursodeoxycholic acid (UDCA), mean dose of 16.7 mg/kg/d; 97.5% of patients (39/40) received UDCA over the course of follow-up. Doses used for both PSC and AIC (respectively, 16.4 and 17.5 mg/kg, *P* = 0.61) were not different. More patients with AIC were treated with immunomodulators (76.9% versus 29.6%, *P* = 0.005) or corticosteroids (84.6% versus 37%, *P* = 0.005). No significant difference was found in the early use of biologics (0% in AIC versus 14.8% in PSC, *P* = 0.12) or overall during the course of follow-up (7.7% in AIC versus 29.6% in PSC, *P* = 0.23). No patients were treated with oral vancomycin.

### Laboratory Follow-Up

One year after diagnosis, both AIC and PSC patients showed a decline in gamma-glutamyl transferase (GGT; both *P* < 0.001), alanine transaminase (ALT; both *P* < 0.001), and aspartate transaminase (AST; both *P* < 0.001) levels. Platelet counts did not change (Fig. 1). However, mean AST, ALT, and GGT levels remained above the upper limit of the normal in AIC, while only mean GGT levels remained elevated in PSC patients (Table 2). Overall, normalization of bilirubin, AST, ALT and GGT levels, and IgG levels for AIC patients at 1 year was achieved in 55.6% of PSC patients (15/27) but only in 15.4% of AIC patients (2/13) (*P* = 0.016). While a decrease in GGT levels of >50% was achieved in 85.2% of PSC cases (23/27) and 92.3% of cases of AIC (12/13), normalization of GGT levels was achieved in 66.7% of PSC (18/27) as opposed to 15.4% of AIC (2/13) (*P* = 0.008).

**TABLE 2.** Laboratory values at 1 year

	Overall	PSC	AIC	*P*
Total bilirubin (µmol/L)	9.0 (6.8)	7.0 (4.2)	13.3 (9.2)	**0.008**
GGT (U/L)	46 (61)	28 (36)	81 (82)	**0.001**
ALP (IU/L)	230	207	277	0.33
AST (U/L)	34 (24)	26 (9)	52 (35)	**0.02**
ALT (U/L)	35(36)	27 (23)	52 (50)	0.08
Platelet counts (× 10^9^/L)	293 (124)	323 (116)	240 (126)	0.21

All values are presented as means (standard deviation) and *P* values are Wilcoxon rank sum tests. Upper limit of normal: Total bilirubin: 17 µmol/L, ALT: 30 U/L, AST: 40 U/L, GGT: 25 U/L, Platelet counts: 150 × 10^9^/L. ALP normal levels varied by age. AIC = autoimmune cholangitis; ALP = alkaline phosphatase; ALT = alanine transaminase; AST = aspartate transaminase; GGT = gamma-glutamyl transferase; PSC = primary sclerosing cholangitis.

Bold indicates significant of *P* < 0.05.

### Long-Term Clinical Follow-Up

**FIGURE 2.**
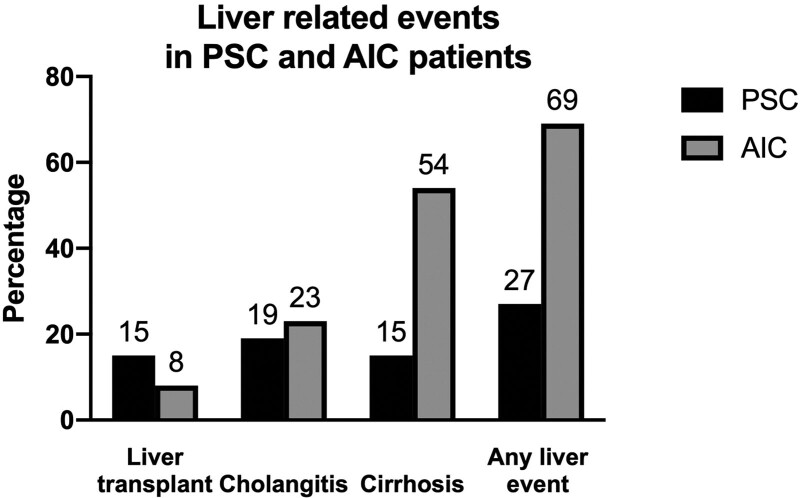
Liver-related clinical events in patients with PSC and AIC. AIC = autoimmune cholangitis; PSC = primary sclerosing cholangitis.

Mean follow-up before transfer to adult hepatology was 56 months (range 2–195 months), with 21 patients (52.5%) benefiting from the extension of follow-up into adulthood. Therefore, mean overall follow-up for the entire cohort was 92 months (range 2–347 months). Biopsy-proven change in diagnosis from PSC to AIC occurred in a single patient. Overall, 5 patients (12.5%) required LT, 11 (27.5%) had a diagnosis of cirrhosis, and 8 (20%) had an episode of acute cholangitis. No deaths or neoplasia (cholangiocarcinoma, hepatocellular carcinoma, or colorectal carcinoma) was recorded over the course of the follow-up. Clinically significant liver-related events were more frequent in patients with AIC than PSC (69.2% versus 39.6%, *P* = 0.02) (Fig. 2).

On Cox proportional hazards regression, a diagnosis of AIC was associated with clinically significant events (hazard ratio [HR] = 3.7 [95% confidence interval (CI): 1.4–10.0] *P* = 0.01) as was baseline bilirubin levels above the upper limit of normal (HR = 5.3 [95% CI: 1.7–16.9] *P* = 0.005). While persistently abnormal liver tests were associated with liver-related events (HR = 8.9 [95% CI: 1.2–67.4] *P* = 0.03), the presence of IBD was not (HR = 0.5 [95% CI: 0.2–1.5] *P* = 0.22). After 1 year, neither normalization of GGT levels (HR = 0.5 (95% CI: 0.2–1.3] *P* = 0.15) nor the decrease of GGT levels by 50% (HR 1.4 [95% CI: 0.3–6.5] *P* = 0.68) was associated with fewer liver-related events. Early use of biologics for treatment of IBD (HR = 1 [95% CI: 0.1–7.8] *P* = 0.99) or overall use of biologic agents (HR = 0.4 [95% CI: 0.1–1.6] *P* = 0.18), as well as thiopurine or corticosteroid use, were not associated with liver-related clinical events.

## DISCUSSION

In this cohort of 40 patients, we explored the long-term evolution of pediatric-onset sclerosing cholangitis into adulthood, contrasting the presentation and outcomes between PSC and AIC. Within this cohort with clearly defined disease phenotypes, AIC patients most often presented with biochemical hepatitis while PSC patients experienced a more favorable biochemical outcome with normalization of laboratory values 1 year postdiagnosis. Over a mean follow-up of 7.7 years and transition into adult care in over 50% of cases, more liver-related events occurred in patients with AIC (HR 3.7).

Establishing a proper diagnosis and stratifying disease severity at baseline remains a challenge in cases of autoimmune liver disease. Consensus diagnostic criteria for AIC are lacking, as the exclusive use of the International Autoimmune Hepatitis Group criteria fails to recognize a potential biliary involvement in children (12,14,15). Whether the natural history of AIC differs significantly from PSC or whether these entities are part of the spectrum of cholestatic liver disease has been previously studied with varying conclusions. The use of serological markers such as autoantibodies and IgG levels can prove useful in making the correct diagnosis. Nonetheless, while these markers are strong predictors of autoimmune hepatitis, determining disease phenotype ultimately relies on histological findings and a low threshold for biopsy is recommended. In our cohort, a change in diagnosis occurred in only one case, highlighting that a thorough evaluation at presentation is essential. The unique clinical course of patients with AIC has previously been described in a prospective study where the progression of biliary disease occurred in 50% of patients with increased levels of fibrosis despite appropriate immunosuppressive treatment. This is surprising since pharmacological therapy was shown to improve inflammation on repeat biopsy (9). This has also been demonstrated in other cohorts where the progression of liver disease and requirement for LT have been found to be more common in AIC (11). Nonetheless, contrary to our findings, other single-center retrospective cohorts have demonstrated a similar rate of liver-related events, including transplant-free survival between AIC and PSC, although the overall follow-up was shorter than in our cohort (6,10,16). Furthermore, in a large multicenter study, AIC phenotype was not associated with altered long-term outcomes; however, small duct disease was linked to favorable outcomes (2). A high rate of advanced fibrosis (beyond 50%) was present at diagnosis in our study population, similar to previous reports from similar sized cohorts (17,18).

The presence of IBD was not associated with liver-related events in our cohort; however, the prevalence of Crohn’s disease was relatively higher than previously reported, potentially reflective of local prevalence and associated with the increased incidence of Crohn’s disease diagnosis over time in the pediatric population (19). While large cohorts have previously reported milder liver disease phenotypes when sclerosing cholangitis was associated with IBD, immunosuppressive regimens were not thoroughly evaluated in these cohorts (2). In our study, use of immunosuppressive agents was evaluated both at the time of diagnosis of liver disease, including agents already in use for IBD, and after the diagnosis of sclerosing cholangitis. Use of biologic agents was not significantly associated with reduced clinical liver events in our cohort, consistent with reports of no improved liver outcomes in pediatric PSC-IBD (20). In addition, use of biologic agents has not been associated with improved liver outcomes in adult PSC (21).

Our study adds to the ongoing discussion whether normalization of liver enzymes is associated with improved outcomes in biliary liver disease. In AIC, disease severity is associated with the presence of portal and lobular inflammation and improvement in this process is required to improve long-term outcomes. Consequently, normalization of liver enzymes is considered as a treatment goal in patients with autoimmune hepatitis and failure to achieve control of inflammation in patients with AIC may lead to increased adverse outcomes. Nonetheless, it remains unclear whether the association observed in AIH between superior outcomes with biochemical and histological normalization applies to AIC as well. In PSC, improvement in biochemical parameters, although imperfect surrogate markers, has been associated with a favorable prognosis. It has been suggested that normalization of GGT levels or significant improvement at 1 year, notwithstanding use of UDCA, is associated with an improved 5-year event-free survival (22). Drastic improvement in liver enzymes in patients with diagnostic criteria for AIC with UDCA treatment alone has been reported (17). However, the true benefit of pharmacological therapy remains controversial as a significant proportion of patients may spontaneous normalize GGT (23). In our cohort, while improvement in liver enzymes over time was associated with favorable outcomes, improvement in GGT alone was not sufficient. Furthermore, while elevation in GGT levels alone at presentation did not differentiate between disease phenotypes, hyperbilirubinemia at baseline was associated with worse clinical outcomes, a stronger predictor than the sclerosing cholangitis clinical phenotype.

Our study is limited by its single-center retrospective design and the small number of patients included. Nonetheless, our study benefits from extensive and long-term follow-up into adulthood, often difficult to achieve without significant dropout in pediatric-onset conditions. This thorough follow-up allows for evaluation of treatment, which has been described in a limited fashion in previous cohorts; however, a causal association between treatment and outcomes could not be inferred due to cohort size. As not all patients included had a complete endoscopic assessment for the presence of IBD, our study is inadequately powered to draw conclusions regarding the impact of subtypes of IBD and the clinical course of intestinal disease on hepatic outcomes. Finally, referral bias remains possible although children with liver disease are routinely referred to specialized pediatric centers with the recommendation of transition of care to an adult hepatology center. Nonetheless, our results show that data from smaller cohorts with well-characterized individual subjects can be relevant to clinical practice.

In a single-center cohort, rigorous phenotyping can potentially outline distinct natural histories for pediatric AIC and PSC. In addition, elevated serum bilirubin levels at diagnosis were associated with liver-related events. Challenges in clinical management remain in order to improve clinical outcomes in sclerosing cholangitis.

## ACKNOWLEDGMENTS

J.H. and F.A. were involved in conceptualization. J.H. and P.W. were involved in data curation and analysis. M.B., C.V., and F.A. were involved in investigation and supervision and project administration. J.H., P.W., and F.A. were involved in writing original draft. All authors were involved in revision of the manuscript for critically important content. All authors approved the final version of the manuscript.

## Supplementary Material


