# Supplementary material for: Long-Term Follow-Up into Adulthood of Pediatric-Onset Primary Sclerosing Cholangitis and Autoimmune Sclerosing Cholangitis
Source: JPGN Rep. 2022 Jun 21;3(3):e220. doi: 10.1097/PG9.0000000000000220 (PMC10158455; doi:10.1097/PG9.0000000000000220)
Supplement: Supplementary file 1 [file pg9-3-e220-s001.pdf]

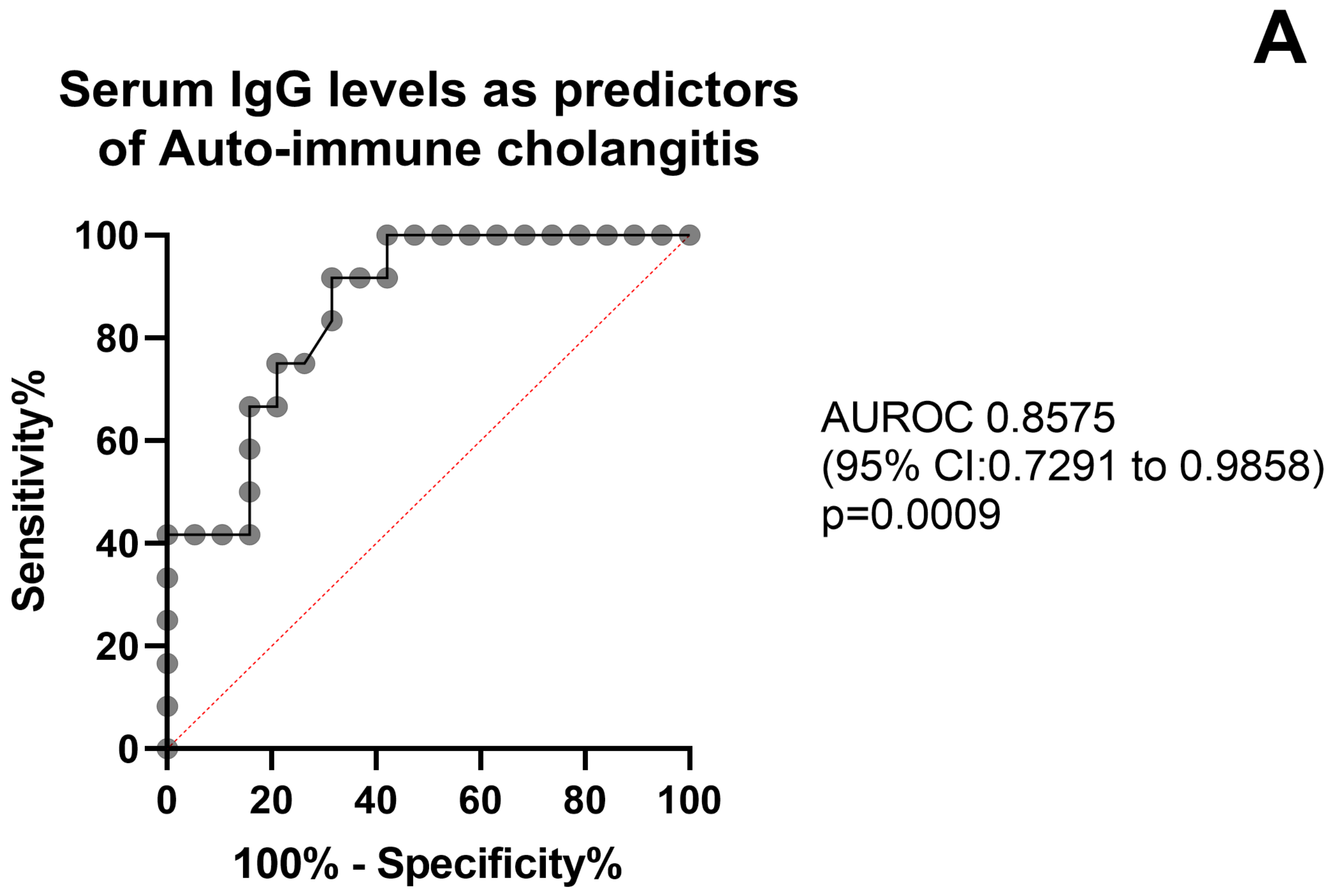
A
Serum IgG levels as predictors of Auto-immune cholangitis
Sensitivity%
100% - Specificity%
0
20
40
60
80
100
AUROC 0.8575
(95% CI:0.7291 to 0.9858)
p=0.0009

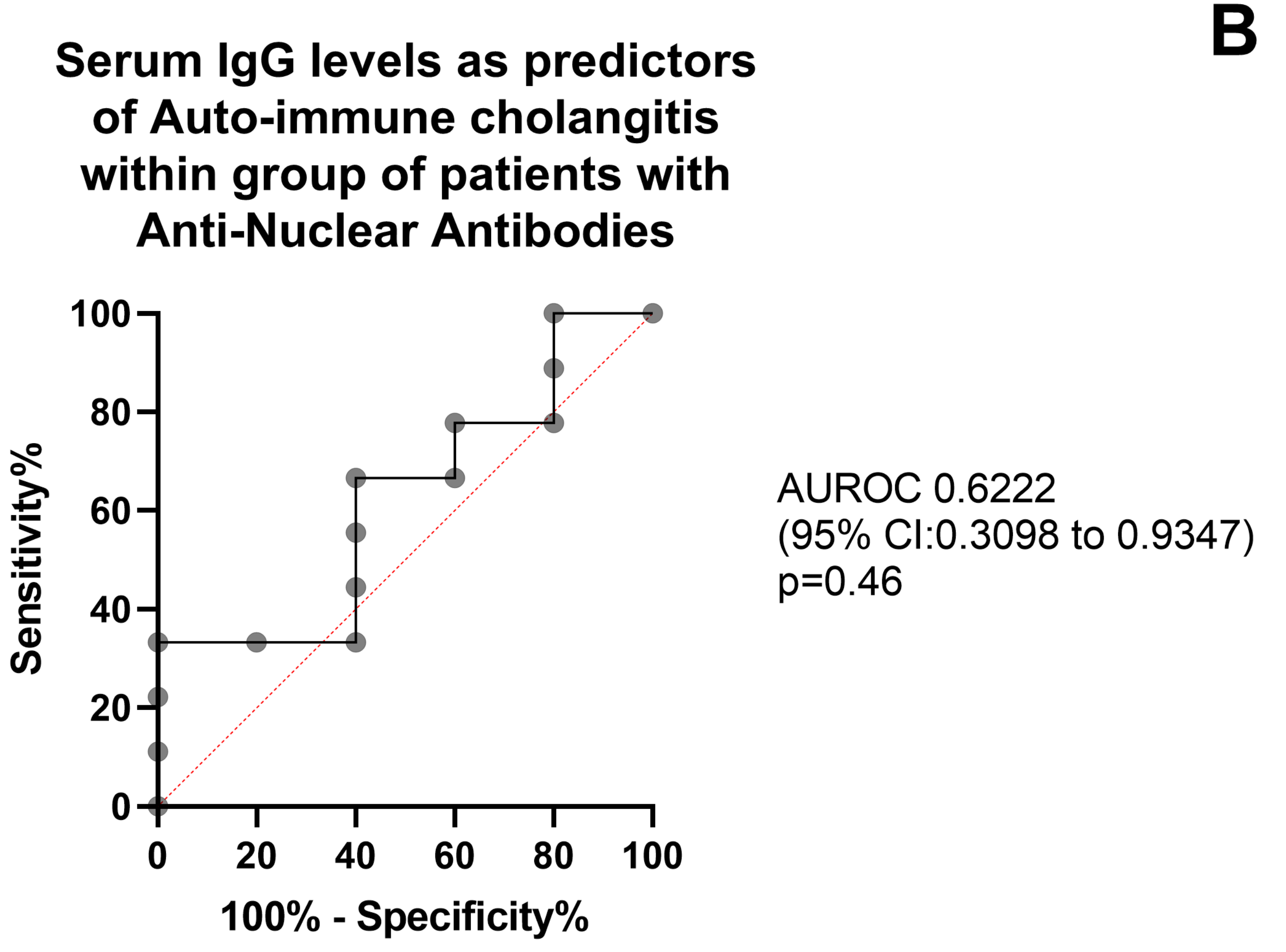
B
Serum IgG levels as predictors of Auto-immune cholangitis within group of patients with Anti-Nuclear Antibodies
Sensitivity%
100% - Specificity%
0
20
40
60
80
100
AUROC 0.6222
(95% CI:0.3098 to 0.9347)
p=0.46
